# Supplementary material for: Delirium symptoms are associated with decline in cognitive function between ages 53 and 69 years: Findings from a British birth cohort study
Source: Alzheimers Dement. 2018 May;14(5):617–22. doi: 10.1016/j.jalz.2017.08.018 (PMC5948100; doi:10.1016/j.jalz.2017.08.018)
Supplement: Supplementary Tables 1 and 2 [file mmc1.docx]

| Patient characteristic | Adjusted for sex | | | | | Adjusted for sex and visual search speed at 53 | | | | Adjusted for all covariates | | | |
| --- | --- | --- | --- | --- | --- | --- | --- | --- | --- | --- | --- | --- | --- |
|  |  |  |  |  |  |  |  |  |  |  |  |  |  |
| Visual Search Speed aged 69 | n | Coefficient | 95% CI | | P>t | Coefficient | 95% CI | | P>t | Coefficient | 95% CI |  | P>t |
| Sex | 1523 | 11 | 4 | 18 | <0.01 | 5 | -2 | 11 | 0.17 | 5 | -1 | 12 | 0.11 |
| Visual Search Speed age 53 (by SD) |  | 36 | 33 | 39 | <0.01 | 36 | 33 | 39 | <0.01 | 35 | 32 | 39 | <0.01 |
| Delirium age 60-69 |  | -32 | -53 | -11 | <0.01 | -33 | -52 | -14 | <0.01 | -31 | -49 | -12 | <0.01 |
| Hypertension at age 60-64 |  | -10 | -17 | -2 | 0.02 | -5 | -11 | 2 | 0.17 | -2 | -9 | 5 | 0.61 |
| Diabetes by 60-64 |  | -5 | -9 | 0 | 0.05 | -5 | -9 | -1 | 0.01 | -4 | -8 | 0 | 0.03 |
| Smoking status at 60-64 |  |  |  |  |  |  |  |  |  |  |  |  |  |
| *Current* |  | Ref |  |  |  | Ref |  |  |  | Ref |  |  |  |
| *Ex* |  | 14 | 1 | 26 |  | 12 | 1 | 23 |  | 9 | -2 | 20 |  |
| *Never* |  | 16 | 3 | 29 | 0.02 | 10 | -1 | 22 | 0.08 | 6 | -5 | 18 | 0.27 |
| Level of activity age at 60-64 |  |  |  |  |  |  |  |  |  |  |  |  |  |
| *Inactive* |  | Ref |  |  |  | Ref |  |  |  | Ref |  |  |  |
| *Less active (1-4/wk)* |  | 9 | -2 | 20 |  | 7 | -3 | 17 |  | 5 | -5 | 15 |  |
| *More active (>5/wk)* |  | 13 | 5 | 22 | <0.01 | 11 | 4 | 18 | <0.01 | 7 | 0 | 15 | 0.14 |
| Educational Attainment by 26 |  |  |  |  |  |  |  |  |  |  |  |  |  |
| *None* |  | Ref |  |  |  | Ref |  |  |  | Ref |  |  |  |
| *O Levels* |  | 7 | -3 | 18 |  | 1 | -9 | 10 |  | -2 | -11 | 7 |  |
| *>=A levels* |  | 21 | 13 | 29 | <0.01 | 9 | 1 | 16 | 0.02 | 5 | -3 | 13 | 0.22 |
| BMI age 60-64 (by SD) |  | -6 | -10 | -2 | <0.01 | -4 | -7 | 0 | 0.03 | -2 | -6 | 1 | 0.18 |

**Supplementary Table 1:** Linear regression analyses of visual search speed by covariates in 1529 study participants with complete covariate data in NSHD cohort, adjusted by sex; additionally by prior visual search speed aged 53; additionally by all covariates

| Patient characteristic | Adjusted for sex | | | | | Adjusted for sex and verbal memory at 53 | | | | Adjusted for all covariates | | | |
| --- | --- | --- | --- | --- | --- | --- | --- | --- | --- | --- | --- | --- | --- |
|  |  |  |  |  |  |  |  |  |  |  |  |  |  |
| Verbal Memory aged 69 | n | Coefficient | 95% CI | | P>t | Coefficient | 95% CI | | P>t | Coefficient | 95% CI | | P>t |
| Sex | 1505 | 1.9 | 1.3 | 2.5 | <0.01 | 0.6 | 0.1 | 1.0 | 0.02 | 0.7 | 0.3 | 1.2 | <0.01 |
| Verbal Memory age 53 (by SD) |  | 4.1 | 3.8 | 4.3 | <0.01 | 4.1 | 3.8 | 4.3 | <0.01 | 3.7 | 3.4 | 3.9 | <0.01 |
| Delirium age 60-69 |  | -0.9 | -2.6 | 0.8 | 0.30 | -0.6 | -1.9 | 0.7 | 0.38 | -0.4 | -1.6 | 0.9 | 0.59 |
| Hypertension at age 60-64 |  | -0.8 | -1.5 | -0.2 | 0.01 | -0.3 | -0.8 | 0.1 | 0.15 | -0.2 | -0.7 | 0.3 | 0.47 |
| Diabetes by 60-64 |  | -0.3 | -0.6 | 0.1 | 0.20 | -0.2 | -0.5 | 0.1 | 0.13 | -0.1 | -0.4 | 0.1 | 0.32 |
| Smoking status at 60-64 |  |  |  |  |  |  |  |  |  |  |  |  |  |
| *Current* |  | Ref |  |  |  | Ref |  |  |  | Ref |  |  |  |
| *Ex* |  | 2.4 | 1.4 | 3.4 |  | 0.7 | 0.0 | 1.5 |  | 0.4 | -0.4 | 1.2 |  |
| *Never* |  | 3.0 | 1.9 | 4.0 | <0.01 | 1.3 | 0.5 | 2.1 | <0.01 | 0.8 | 0.0 | 1.7 | 0.08 |
| Level of activity age at 60-64 |  |  |  |  |  |  |  |  |  |  |  |  |  |
| *Inactive* |  | Ref |  |  |  | Ref |  |  |  | Ref |  |  |  |
| *Less active (1-4/wk)* |  | 2.3 | 1.4 | 3.2 |  | 1.2 | 0.6 | 1.9 |  | 1.0 | 0.3 | 1.7 |  |
| *More active (>5/wk)* |  | 2.2 | 1.6 | 2.9 | <0.01 | 0.9 | 0.4 | 1.4 | <0.01 | 0.6 | 0.1 | 1.1 | <0.01 |
| Educational Attainment by 26 |  |  |  |  |  |  |  |  |  |  |  |  |  |
| *<O Levels* |  | Ref |  |  |  |  |  |  |  | Ref |  |  |  |
| O Levels |  | 4.3 | 3.6 | 5.1 |  | Ref | 0.6 | 1.9 |  | 1.1 | 0.5 | 1.8 |  |
| *>=A Levels* |  | 5.4 | 4.7 | 6.0 | <0.01 | 1.8 | 1.2 | 2.3 | <0.01 | 1.5 | 0.9 | 2.1 | <0.01 |
| BMI age 60-64 (by SD) |  | -0.6 | -0.9 | -0.2 | <0.01 | -0.3 | -0.5 | -0.1 | 0.02 | -0.1 | -0.4 | 0.1 | 0.27 |

**Supplementary Table 2:** Linear regression analyses of verbal memory by covariates in 1511 study participants with complete covariate data in NSHD cohort, adjusted by sex; additionally by prior visual search speed aged 53; additionally by all covariates
